# An Intraoral OCT Probe to Enhanced Detection of Approximal Carious Lesions and Assessment of Restorations

**DOI:** 10.3390/jcm9103257

**Published:** 2020-10-12

**Authors:** Hartmut Schneider, Martin Ahrens, Michaela Strumpski, Claudia Rüger, Matthias Häfer, Gereon Hüttmann, Dirk Theisen-Kunde, Hinnerk Schulz-Hildebrandt, Rainer Haak

**Affiliations:** 1 Department of Cariology, Endodontology and Periodontology, University of Leipzig, 04103 Leipzig, Germany; michaela.strumpski@medizin.uni-leipzig.de (M.S.); claudia.rueger@medizin.uni-leipzig.de (C.R.); matthias.haefer@medizin.uni-leipzig.de (M.H.); rainer.haak@medizin.uni-leipzig.de (R.H.); 2 Institut für Biomedizinische Optik, Universität zu Lübeck, 23562 Lübeck, Germany; ahrens@bmo.uni-luebeck.de (M.A.); huettmann@bmo.uni-luebeck.de (G.H.); hinnerk.schulz-hildebrandt@mll.uni-luebeck.de (H.S.-H.); 3 Medizinisches Laserzentrum Lübeck GmbH, 23562 Lübeck, Germany; dirk.theisenkunde@uni-luebeck.de; 4 Airway Research Center North (ARCN), German Center for Lung Research (DZL), 23562 Lübeck, Germany

**Keywords:** OCT, intraoral probe, carious lesions, caries diagnosis, dental restorations

## Abstract

Caries, the world’s most common chronic disease, remains a major cause of invasive restorative dental treatment. To take advantage of the diagnostic potential of optical coherence tomography (OCT) in contemporary dental prevention and treatment, an intraorally applicable spectral-domain OCT probe has been developed based on an OCT hand-held scanner equipped with a rigid 90°-optics endoscope. The probe was verified in vitro. In vivo, all tooth surfaces could be imaged with the OCT probe, except the vestibular surfaces of third molars and the proximal surface sections of molars within a "blind spot" at a distance greater than 2.5 mm from the tooth surface. Proximal surfaces of 64 posterior teeth of four volunteers were assessed by intraoral OCT, visual-tactile inspection, bitewing radiography and fiber-optic transillumination. The agreement in detecting healthy and carious surfaces varied greatly between OCT and established methods (18.2–94.7%), whereby the established methods could always be supplemented by OCT. Direct and indirect composite and ceramic restorations with inherent imperfections and failures of the tooth-restoration bond were imaged and qualitatively evaluated. The intraoral OCT probe proved to be a powerful technological approach for the non-invasive imaging of healthy and carious hard tooth tissues and gingiva as well as tooth-colored restorations.

## 1. Introduction

One of the main causes for dental care and especially invasive restorative treatments is still dental caries as the most common chronic disease worldwide [1,2]. It is only a paradigm change in the treatment of dental diseases [3] that will cause a shift in the focus of dental considerations towards more prevention and evidence-based caries management in order to inhibit or prevent this multifactorial disease. This requires a more reliable detection of carious lesions at an early stage so that they can be treated in time or with a non- or minimally invasive approach [4,5]. As standard diagnostic methods, visual-tactile inspection, even if it differentiates between various categories, and X-ray diagnosis are insufficient to detect carious lesions in an early state [6,7,8,9,10]. In particular, when considering all possible study populations, their reliable detection in fissures (hidden caries) or the proximal surfaces of posterior teeth is not provided [11]. This applies in particular when the depth of the lesion or the presence of proximal micro-cavitations has to be assessed [12,13]. In addition, the use of X-rays leads to harmful radiation exposure of patients. Since non- or minimally invasive treatment options are becoming increasingly important [3], this results in unique challenges for caries diagnostics. In particular, it should also be possible to monitor the lesions after treatment without radiation exposure as often as necessary to control the success of it or the development of the lesions. In recent years, numerous innovative caries detection methods have been introduced, based for example on fluorescence techniques [4,14], impedance measurement, near-infrared light transillumination [13,15] or fiber-optic transillumination (FOTI) [10,11,12]. However, these methods only partially fulfill the highly specific requirements placed on today’s caries diagnosis [8,16].

The same considerations have to be made when evaluating tooth-colored restorations. The integrity of the bond between tooth and restoration and imperfections or defects in restoration materials affect their clinical success. If bacteria migrate into the margin gaps of restorations, this can cause carious lesions adjacent to the restoration (secondary caries) [17]. Secondary caries or its mere assumption, e.g., due to marginal discoloration, is one of the most frequent causes for the replacement of fillings today.

The longitudinal assessment of lesion activity by monitoring the progression is another important aspect of assessing restorations and arrested lesions might also have a positive prognosis due to repair processes [18]. The clinical evaluation of restorations is still based on the visual-tactile inspection of the filling surfaces and restoration margins [19], sometimes combined with radiological evaluation or, in randomized clinical studies, with the more extensive quantitative marginal analysis [20]. However, quantitative marginal analysis does not provide information about the quality of internal adaptation, nor can the filling be analyzed immediately after placement in these clinically inaccessible areas. Since there are no standardized diagnostic criteria in clinical routine, a large number of false diagnoses and thus frequent overtreatment can be assumed [18].

In the past, numerous studies have shown the potential of the image-based, non-invasive optical coherence tomography (OCT) in dental applications, including caries diagnosis and the evaluation of tooth-colored restorations [21,22,23,24,25,26,27]. Introduced in 1991 in medicine [28], it is routinely used in ophthalmology for early detection of pathological changes at the retina and optic nerve [29]. Further applications can be found, for example, in dermatology [30] and cardiology [31].

The cross-sectional images generated by OCT in 3D image stacks are based on the different scattering and absorption of light at the structures of the hard tooth tissues and oral soft tissue. The structures are imaged with high spatial resolution in the µm range and high sensitivity. Their qualitative and quantitative evaluation is possible. The fast generation of images allows real-time imaging, which enables the intraoral application of OCT. First, intraoral images of the human hard tooth and oral soft tissues were taken in 1998 with an OCT hand-held probe at the central wavelength of 1310 nm. The lateral resolution was d_x_ = 50 µm and the axial resolution d_z_ = 20 µm [32]. Some in vivo applications of OCT followed with special hand-held probes to also image carious or non-carious lesions [33,34,35,36,37] or soft tissue [38] and to evaluate composite restorations using a standard device in long-term clinical studies [20,24,25]. In particular, the combination of clinical evaluation of composite restorations and the OCT assessment of the tooth-composite bond failure showed that OCT could provide important additional diagnostic information. The hand-held OCT scanners presented so far in the literature differ among other things in the type of OCT, the light wavelengths and bandwidths used, the power on sample, or the design.

An intraoral OCT probe was developed based on a spectral-domain (SD)-OCT system proven in in-vitro and in-vivo studies [20,23,24,25]. The focus was on a high spatial resolution, high signal-to-noise ratio at low power on sample and suitable ergonomics for performing tomography of the whole dentition from occlusal, vestibular and oral positions. In this study, the use of the probe for the detection of proximal carious lesions in posterior teeth is presented in comparison to visual-tactile evaluation, X-ray diagnosis and FOTI. Additionally, the potential for imaging natural structures of human teeth and tooth-colored restorations is shown. The hypotheses were that (1) the intraorally applicable OCT probe can be used to display internal anatomical structures of human hard tooth tissues, (2) OCT and established methods differ in the detecting proximal carious lesions, (3) the OCT probe is a reliable complement to the established methods for proximal caries diagnosis, and (4) relevant additional information is generated to evaluate indirect composite and ceramic restorations.

## 2. Materials and Methods

### 2.1. SD-OCT System and Hand-Held Probe

**Figure 1:**
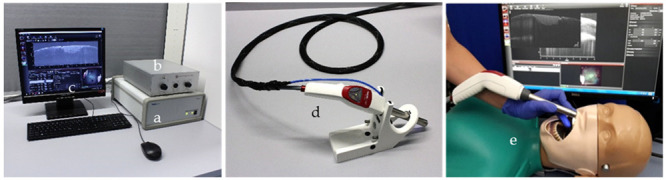
Images of the complete set-up including the OCT system (**a**), custom-designed reference arm (**b**), peripheral equipment of the PC (**c**), and the modified hand-held scanner (**d**). To evaluate and optimize the handling of the hand-held scanner, a patient equivalent simulation (**e**) was used to perform test measurements on artificial sets of teeth and, in addition, also on extracted human teeth.

The system set-up is shown in Figure 1. A spectral-domain OCT system Telesto-II SP 2 with an external reference and a hand-held scanner OCTH-1300 (Thorlabs GmbH, Dachau, Germany) using the central wavelength 1310 nm was supplemented by an endoscopic probe (Medizinisches Laserzentrum Lübeck GmbH, Lübeck, Germany). The axial resolution of the system and the imaging depth in hard tooth tissue are 5.5 µm (water) and 2.5 mm [37]. The endoscope optics reaches a lateral resolution of 11.5 µm and a lateral field of view 8 × 8 mm. The power on sample is 2.3 mW. A fiber coupler with a splitting ratio of 50:50 divides the light into a reference and a sample arm. The reference arm comprises a fiber optical collimator to parallelize the light, an iris diaphragm to adjust the power, and a retroreflector to reflect the light into the fiber. The intraoral OCT probe, attached to the sample arm, consists of a modified MEMS hand-held scanner (OCTH-1300NR, Thorlabs GmbH, Dachau), in which the internal reference was blocked, and the objective was replaced by a custom build endoscope (Medizinisches Laserzentrum Lübeck GmbH).

**Figure 2:**
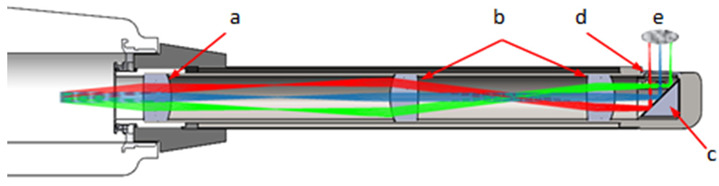
Simulated beamline of the intraoral probe with scan lens (**a**), relay-optics (**b**), silver-coated right-angle prism mirror (**c**), glass plate (**d**), and focal area (**e**).

The endoscope optics was designed using OpticStudio (Zemax, LLC, Kirkland, WA, USA). It consists of a scan lens (OCTH-LK30, Thorlabs GmbH, Dachau), a relay optic (4f-optic) (2x #45-793 Edmund Optics Inc., Barrington, IL, USA) and a silver-coated right-angle prism mirror (MRA10-P01, Thorlabs GmbH, Dachau). The optical components were placed in a stainless-steel tube (Harry Rieck Edelstahl GmbH, Hilden, Germany). The tube has a diameter of 16 mm and a length of 110 mm. A glass plate was used as an end window of the endoscope (Figure 2). In Table 1, the parameters of the whole system are shown.

**Figure 3:**
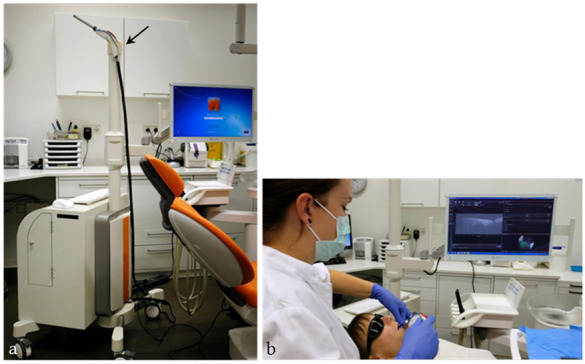
The system set-up with the intraoral OCT probe ((**a**), arrow) permits chairside the intraoral diagnosis in communication with the patient (**b**).

Based on the comparison with OCT-B-scans on individual teeth taken with the standard probe of the Telesto (OCTG-1300, Thorlabs GmbH, Dachau), the intraoral device and the image display were technically optimized (oral cavity illumination, signal amplification, disinfection of the probe). A footswitch for starting and stopping recording (2D, 3D) has been connected. The system set-up with the computer hardware and a monitor for diagnostic purposes (MX-22, Neovo, Capelle a. d. Ijssel, Netherlands) was mounted on a mobile custom build card system (orangedental GmbH & Co. KG, Biberach a. d. Riß, Germany) and covered for chairside use according to Medical Device Regulation (MDR, EU 2017/745) (Figure 3).

### 2.2. In-Vitro Study

The study was conducted in accordance with the Declaration of Helsinki, and the protocol was approved by the Ethics Committee of the University of Leipzig. The extracted teeth were used based on ’patients’ approvals (informed consents, protocol no. 299-10-04102010).

Twenty extracted human healthy or carious anterior and posterior teeth were used. The teeth were unrestored or restored with composite, were arranged in C-silicone (Henry Schein Inc., Melville, NY, USA) according to their natural arrangement, and fixed in a patient-equivalent simulation. With the probe, the hard tooth tissues, approximal carious lesions, and tooth-colored restorations were visualized in OCT cross-sectional images with a maximum field of view 8 mm × 8 mm × 3.5 mm (air, maximum pixel size 800 × 400 × 1024). The probe was mechanically stabilized by hand support and placed at a distance of approximately 1 cm from the tooth surface (Supplementary Materials Figure S1).

The signals for cavitated or non-cavitated proximal lesions or interfacial adhesive defects in composite restorations, imperfections in restoration materials and structural defects in hard tooth tissues that could be imaged in the OCT-B scans were verified by x-ray microtomography (Skyscan 1172-100-50, Bruker MicroCT, Kontich, Belgium) and light microscopy (unsectioned/sectioned specimens; Stemi 2000-C, Carl Zeiss Microscopy GmbH, Jena, Germany) [23,39,40,41,42].

### 2.3. In-Vivo Study Caries Diagnosis

#### 2.3.1. Visual-Tactile Probing/FOTI/Radiography/Intraoral OCT

The study was conducted in accordance with the Declaration of Helsinki and the protocol was approved by the Ethics Committee (see above). All subjects gave their informed consent for admission before participating in the study. Four volunteers were recruited from the probe development group (age 24–65 years, Department of Cariology, Endodontology and Periodontology, University of Leipzig). Inclusion criteria were: no orthodontic treatment with fixed appliances, no congenital tooth anomalies, no limitation in mouth opening, and no pregnancy.

Sixty-four approximal surfaces of the posterior teeth of the four volunteers with unrestored proximal healthy regions and carious lesions have been included in the study (Supplementary Materials Figure S1). At a dental unit (TENEO, Sirona Dental Systems GmbH, Bensheim, Germany) under standardized conditions (operating light and standardized room lighting), the teeth were cleaned with Nupro Sensodyne Stain Removal and Nupro Sensodyne Polish (Dentsply Sirona Deutschland GmbH, Bensheim, Germany) and air-dried with compressed air. The proximal areas were photographed from occlusal (Nikon D7000, Nikon AF-S NIKKOR 105 mm, Nikon GmbH, Düsseldorf, Germany; Nissin MF18 Macro Flash, Nissin Japan Ltd., Tokyo, Japan). The dry proximal surfaces were visually inspected according to ICDAS [43] by an experienced clinician (M. S.).

Subsequently, the proximal areas were transilluminated both orally and vestibularly with the operating light switched off (FOTI-DIA-STICK, white light; I. C. Lercher GmbH & Co. KG, Stockach, Germany) and captured again with the digital camera. Digital bitewing radiographs were taken with a Heliodent DS Dental X-ray unit using the paralleling technique (7 mA, 60 kV, Dentsply Sirona Deutschland GmbH, Bensheim, Germany; bitewing film holder, Kentzler-Kaschner Dental GmbH, Ellwangen/Jagst, Germany; image plate, sensor size 3 × 4 cm, Dürr Dental AG, Bietigheim-Bissingen, Germany). The images were scanned with the VistaScan Mini Plus (Dürr Dental AG Bietigheim-Bissingen, Germany) and analyzed using DBSWin software (version 5.15.1, Dürr Dental AG, Bietigheim-Bissingen, Germany).

Using the hand-held OCT probe, the tooth surfaces were imaged according to the parameters in Table 1, whereby the probe was mechanically stabilized by hand or with a silicone impression on the opposite jaw. Within the field of view, two-dimensional image sequences were generated from occlusal, vestibular, or oral view and by varying the angle of incidence of the OCT probe.

#### 2.3.2. Calibration of Examiners and Image Analysis

Two evaluators (H. S., C. R.) experienced in the assessment of OCT-B scans calibrated a cariologically-experienced examiner (M. S.) that performed the clinical examination and made the FOTI images, X-rays, and OCT-B scans. Prior to the start of the study, the operator was trained in the handling of the probe for imaging surfaces on isolated teeth as well as on those in sets of teeth in the patient-equivalent simulation. After blinding the photographs, the radiographs, the FOTI images, and the OCT-B scans, they were evaluated by two clinicians (M. S; F. K.) in a joint session and arbitrary order using a high-resolution monitor (Color Edge CG 248, EIZO, Hakusan, Japan) certified for X-ray diagnosis. From the OCT image stacks, those 2D images were selected which showed the greatest extent of the lesions. In case of disagreement between the findings of the two examiners, an agreement was subsequently reached by consensus.

#### 2.3.3. Scoring and Statistical Analysis

The evaluation of healthy and carious regions was carried out with the four methods as follows:

- Visual inspection: ICDAS, sound (0, ICDAS, code 0) or carious lesion (1, ICDAS, code 1–4)
- Fiber optic transillumination: no shadow (0), shadow representing a carious lesion (1)
- Bitewing radiography: no radiolucency (0) or radiolucency (1)
- OCT: B scans, no signal representing a carious lesion (0) or signal present (1)

Contingency tables were used to descriptively analyze the relationship between two methods in terms of their scores 0 (sound) and 1 (carious lesion). The four methods were statistically compared by Friedman and McNemar tests. The significance level was set to α = 0.05 (IBM SPSS Statistics 25 for Windows; IBM Corp., Armonk, NY, USA).

By triple reassessment of 27 healthy and carious tooth surfaces of two volunteers at intervals of 7 to 14 days, the intra-rater reliability was determined (average percentage agreement of scores) for scoring according to visual inspection, FOTI, bitewing radiography, and OCT. The visual evaluation was completely reproducible (100.0%), followed by OCT (89.8 %, high agreement), FOTI (76.6%) and radiography (55.4%, low agreement).

### 2.4. In-Vivo Study-Assessment of Tooth-Colored Restorations

In ten volunteers of the probe development group, on anterior and posterior teeth, ten composite restorations (Class I, II, V), composite inlays, and ceramic crones were imaged intraorally with the hand-held OCT probe. The hard tooth tissues with inherent structural defects were displayed. On restored teeth, the sealing of the cavity surfaces with adhesive, the tooth-restoration bond, interfacial adhesive defects or imperfections in the restorations were qualitatively evaluated.

## 3. Results

### 3.1. OCT Imaging of Human Teeth In Vitro and Intraorally

**Figure 4:**
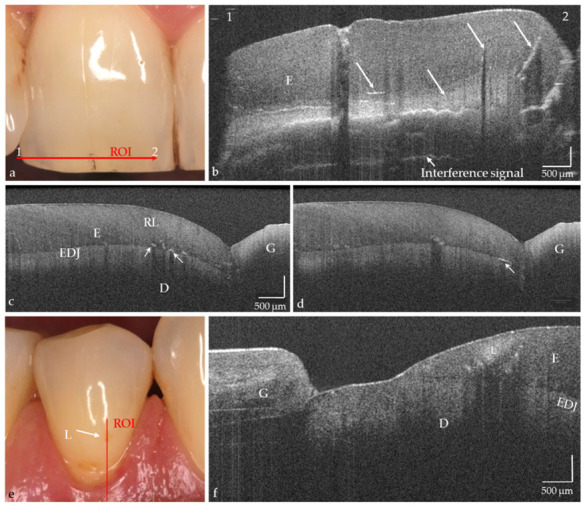
In vivo imaging of hard tooth tissues (**a**–**f**), intrinsic structures, and defects in enamel ((**b**–**d**), arrows). Partially also Retzius lines (RL; (**c**)) and superficial tissue structures of the gingiva (**f**) are indicated. (**e**) Premolar with vestibular brown discoloration. The OCT cross-sectional image (**f**) showed that besides the discoloration a carious lesion in enamel (L) is present. The lesion body appears as a bright zone with clear shadowing. Scales are related to refractive index *n* = 1.0. While the horizontal scale in an OCT cross-sectional image is independent of the refractive index (n) of the tooth structures, the length of the vertical scale has to be divided by it (mean n for enamel and dentin approx. 1.5). *Enamel (E), dentin (D), enamel-dentin junction (EDJ), gingiva (G), region of interest (ROI).*

**Figure 5:**
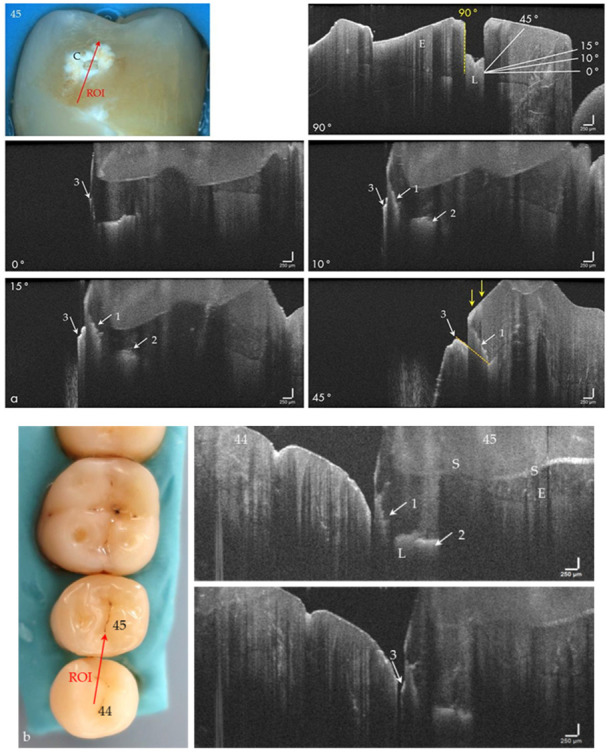
(**a**) Extracted tooth 45 with a cavitated approximal enamel lesion (C). In the OCT image, recorded perpendicular (90°) to the surface, the lesion (L) appeared as a box-shaped structure in the enamel. If the cavitation is imaged from occlusal with an angle of incidence of 0° to 45°, the representation changes. The upper cavity wall appears as a diagonal signal line (arrows 1). Furthermore, the lesion body (arrows 2) and the cervical margin of the cavitation become visible (arrows 3). (**b**) Artificial row of teeth. In the ROI, the diagonal signal of the cavity (arrow 1) merges into a horizontal signal line representing the lesion body extending to the enamel-dentin junction (arrow 2). With a slight variation of the probe position, the protruding edge of the cavity entrance opening appears at some point (arrow 3). *When focusing on deeper structures, the tooth surface (S) is inverted in the OCT image. The vertical scales are related to refractive index n = 1.0 (see the remark in Figure 4). Enamel (E).*

The hard tooth tissues enamel, dentin, and cement, including intrinsic structures such as Retzius lines or defects, could be imaged both on the phantom head and intraorally (Figure 4a–d, in vivo). The cross-sectional OCT images were comparable to those obtained with the laboratory probe. Early carious lesions and their dimensions were displayed in vitro and in vivo, whereby discolorations and demineralized zones could also be distinguished (Figure 4e,f). Micro-cavitations in the enamel could be verified in vitro (Figure 5).

**Figure 6:**
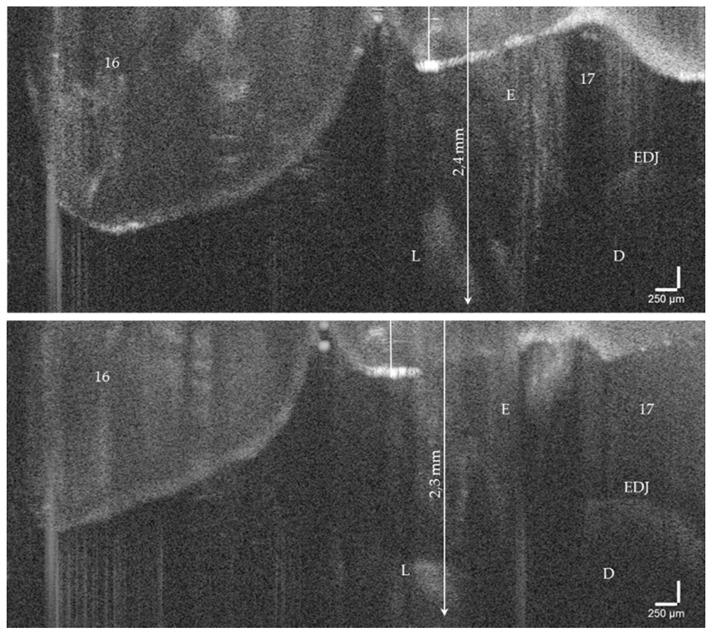
OCT cross-sectional image of an early approximal enamel carious lesion (L; ICDAS, Code 2) on tooth 17 in vivo. The lesion can be localized to a depth of 2.4 mm below the enamel surface. *As a result of focusing on deeper structures, the tooth surface is flipped over again. Enamel (E), dentin (D), enamel-dentin junction (EDJ). The vertical scales are related to refractive index n = 1.0 (see the remark in Figure 4).*

On hard tooth tissues, the imaging depth was up to 2.5 mm (Figure 6). The spatial resolution of the probe also partially allowed the visualization of superficial tissue structures of the gingiva (Figure 4e,f). The 90°-optics and the ergonometric design of the probe allowed the dentist to manually image all occlusal, vestibular, oral, and proximal tooth surfaces including those of the second molars and the occlusal, oral and proximal surfaces of the third molars. The vestibular surfaces of third molars and proximal surface areas of the molars within a “blind spot” at a distance greater than 2.5 mm from the tooth surfaces could not be imaged. While a B-scan is recorded in real time, the scan time for a C-scan is approximately 28 seconds.

### 3.2. Approximal Caries Diagnosis

Sixty-four proximal tooth surfaces of premolars and molars differing from healthy to cavitated were examined by visual inspection, FOTI, bitewing radiographs and intraoral OCT. The lesions were located above, at or below the contact point, and sometimes adjacent to metallic and non-metallic restorations.

**Figure 7:**
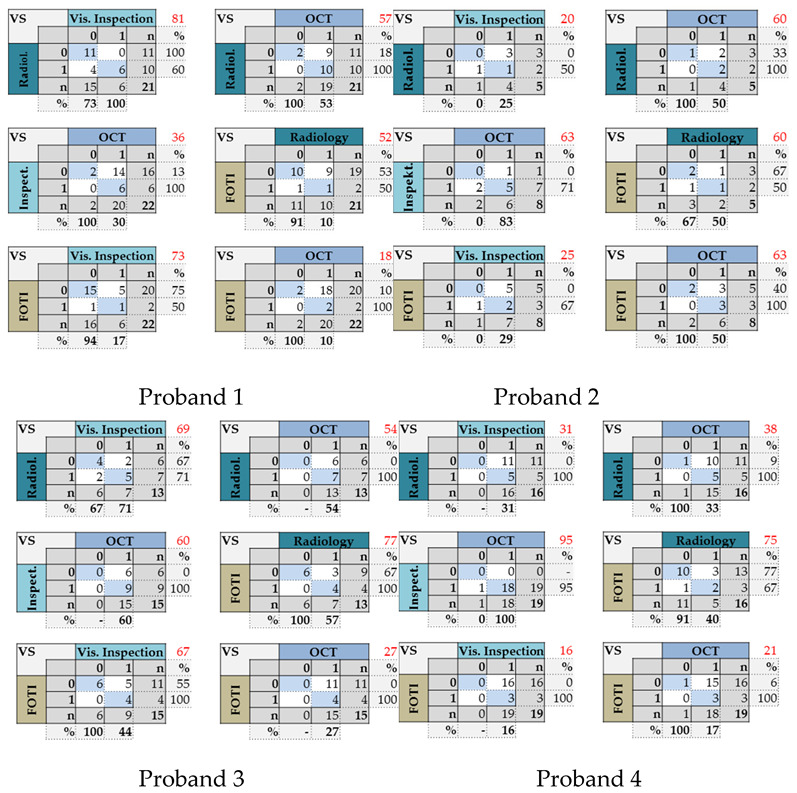
Contingency tables. Pairwise comparison of the four caries detection methods. The red marked percentages indicate the agreement of two methods for both scores 0 and 1. There were fewer proximal areas that could be evaluated with radiography, as some could not be assessed due to superimpositions.

**Figure 8:**
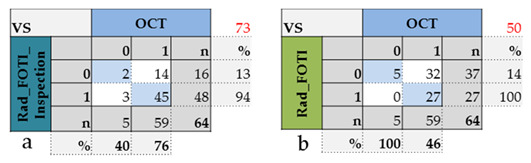
Contingency tables. Highest diagnostic score 1 resulting from the introduced methods radiology/fiber-optic transillumination (FOTI)/visual inspection (**a**) or from radiology/FOTI (**b**) compared to OCT (64 surfaces).

**Figure 9:**
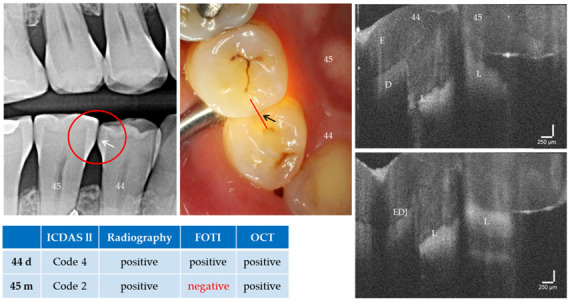
Teeth 44 (distal) and 45 (mesial). Detection of approximal caries lesions (L) with visual inspection, bitewing radiology, FOTI and OCT. The distal surface of tooth 44 was assessed as carious by all methods (arrows, L). In contrast, tooth 45 mesially was assessed as being sound with FOTI. In the OCT cross-sectional images the carious lesions are clearly visible on both teeth. Tooth 44 (ROI) shows no dentin involvement in the upper OCT image, whereas this can be suspected in the lower image. Both tooth surfaces showed no cavitation even with variable positioning of the OCT probe. *As a result of focusing on deeper structures, the tooth surface has flipped in the OCT images. Enamel (E), dentin (D), enamel-dentin junction (EDJ). The vertical scales are related to the refractive index n = 1.0 (see remark in Figure 4).*

**Figure 10:**
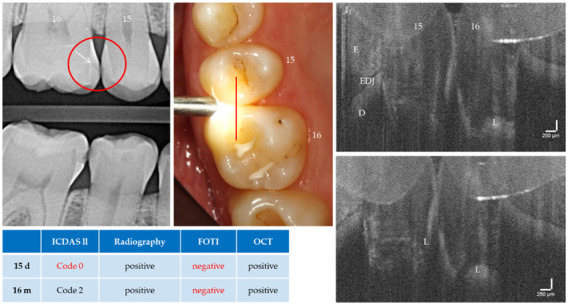
Teeth 16 (m) and 15 (d). The OCT detects a cavitated carious dentin lesion (L) on tooth 16 mesially as a diagonal signal line (see Figure 5b). In contrast, the distal surface of tooth 15 appears demineralized and intact. Both lesions can be detected radiologically (arrow) but not with FOTI. Tooth 15 distal showed no visual evidence of a lesion. *As a result of focusing on deeper structures, the tooth surface has flipped in the OCT images. Enamel (E), dentin (D), enamel-dentin junction (EDJ). The vertical scales are related to the refractive index n = 1.0 (see remark in Figure 4).*

**Figure 11:**
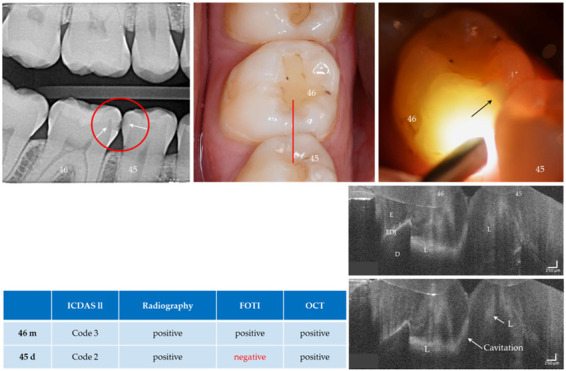
Teeth 46 (m) and 45 (d). Both surfaces showed visual and radiographic caries lesions (L, arrows) and dentin involvement was visible on tooth 46. The FOTI showed no signal on tooth 45 distally (arrow indicates the lesion of tooth 46), while a weak signal was visible on OCT. In the OCT images of tooth 46, dentin involvement is clearly visible and cavitation can be assumed due to the diagonal signal line (see Fig. 5b) and the anatomy of the tooth surface. *Verification*: Both the early carious lesion on tooth 45 and the cavitated lesion on tooth 46 could be confirmed later during the restoration of tooth 46. *Enamel (E), dentin (D), enamel-dentin junction (EDJ). The vertical scales are related to refractive index n = 1.5*.

**Figure 12:**
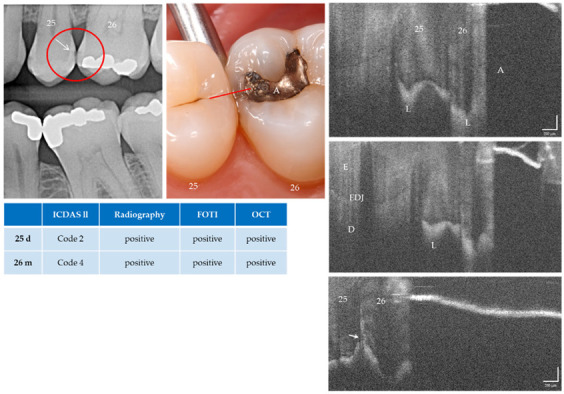
Teeth 25 (d) and 26 (m). All methods diagnosed enamel caries lesions (L, arrow). On tooth 26 mesial the lesion was located next to an amalgam filling (A). The lower OCT image shows an irregularity in the enamel surface (arrow). An extensive lesion became visible by varying the angle of light incidence. Above the lesion the structures in the enamel are clearly visible. Whether the inclined signal in the surface of tooth 25 is a cavitation cannot be determined with certainty. Further OCT images taken orally and vestibularly did not provide clear additional information. Dentin involvement of the lesion is suspected, but an exact assessment is not possible due to local shadowing. *As a result of focusing on deeper structures, the tooth surface has flipped in the OCT images. Enamel (E), dentin (D), enamel-dentin junction (EDJ). The vertical scales are related to the refractive index n = 1.0 (see remark in Figure 4)*.

**Figure 13:**
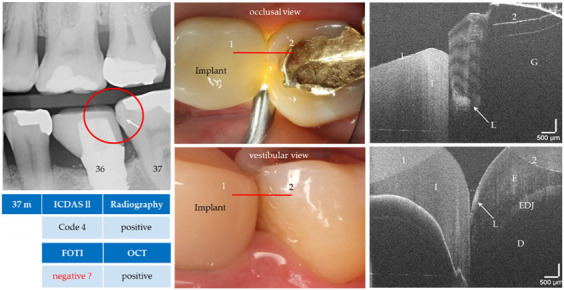
Tooth 37 (m). Apart from the FOTI, the enamel lesion (L, arrows) next to a gold inlay (G) was detected visually, radiographically and by OCT from both the occlusal and vestibular position. The proband was also able to confirm that a non-cavitated lesion had been detected before insertion of the implant crown (I, e.max CAD). The red lines mark the OCT cross-sectional planes. *As a result of focusing on deeper structures, the tooth surface has flipped in the OCT images. Enamel (E), dentin (D), enamel-dentin junction (EDJ). The vertical scales are related to the refractive index n = 1.0 (see remark in Figure 4).*

With the OCT, early approximal caries lesions (ICDAS, codes 0–2) could be reliably visualized (Figure 6, Figure 7, Figure 8, Figure 9, Figure 10, Figure 11 and Figure 12), but also advanced lesions with and without micro-cavitation reaching up to the dentin (Figure 9, Figure 10, Figure 11, Figure 12 and Figure 13). The figures use examples to illustrate what diagnostic information can or cannot be obtained with the individual methods on healthy or carious tooth surfaces and how the techniques can complement each other. Since no reference method is available in vivo, the relationships between the diagnostic methods for each proband were presented in contingency tables (Figure 7). The diagonally arranged values illustrate the numerical agreement of a pair of methods for both values 0 and 1 (percent agreement red). The percentages in the outer columns or rows show the frequencies (%) with which both methods match in the row or column with respect to the scores 0 and 1.

Considering all four probands, the agreement between the four methods varied extensively from 16% to 95%. The agreement between OCT and visual inspection differed between 36.4% and 94.7%, between OCT and radiography from 37.5% to 60.0% and between OCT and FOTI in the range 18.2% to 62.5%. Further comparisons showed the following: radiography—FOTI 52.4% to 76.9%, visual inspection—radiography 20.0% to 80.9% and visual inspection—FOTI 15.8% to 72.7%. In contrast, using the highest diagnostic score of one of the three introduced methods resulted in a high agreement with OCT of 73.4% (Figure 8, 64 surfaces).

In all probands, numerous areas were assessed as healthy (score 0) with the established procedures, but showed lesions (score 1) when OCT was added (Figure 7). In contrast, the reverse effect appeared only three times (probands 2 and 4, OCT, visual inspection). While 7.7% of the surfaces with OCT were considered sound (weighted average of four persons, Figure 7), visual inspection, radiography, and FOTI resulted in higher values of 36.5%, 53.0%, and 81.0%, respectively. This phenomenon is also evident in Figure 8.

Figure 7 also shows that the differences between the methods depend on the subject. Compared to visual inspection, OCT differed significantly in two of the four probands (*p*_1,3,2,4_: < 0.001/0.031/0.500/1.000). Radiography or FOTI were different to OCT in three out of the four probands (*p*_radio__4,1,3,2_: 0.002/0.004/0.016/0.500; *p*_foti__1,4,3,2_: < 0.001/< 0.001/0.001/0.250). In contrast, visual inspection vs. radiography or FOTI (*p*_4,4_ < 0.001) and radiography vs. FOTI (*p*_1_ = 0.021) were significantly different in one out of the four probands.

Cavitations in enamel were not quantified in this study, and the methods have not been evaluated comparatively in this respect.

### 3.3. Tooth-Colored Restorations—Intraoral Imaging and Evaluation

With the OCT probe, eight available composite restorations, one ceramic restoration (Figure 13), as well as one composite inlay, could be imaged on anterior and posterior teeth.

**Figure 14:**
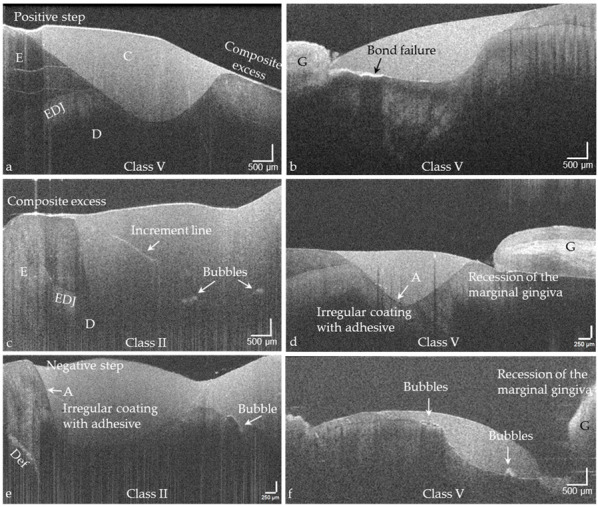
Exemplary OCT images of structural features in Class II and V composite restorations (C) that determine their functionality and are essential for the evaluation of the restorations. (**a**) Positive step and composite excess at the restoration margins, (**b**) interfacial adhesive defect (bond failure), (**c**) composite excess at the restoration margin, increment lines and bubbles in the restoration material, (**d**) irregular coating of the cavity with adhesive (A) and recession of the marginal gingiva (G), (**e**) negative step at a restoration margin, irregular coating with adhesive and bubble in the restoration material, (**f**) bubbles in the restoration and recession of the marginal gingiva. *Enamel (E), dentin (D), the enamel-dentin junction (EDJ), cohesive defect (Def). The vertical scales are related to the refractive index n = 1.0 (see remark in Figure 4).*

**Figure 15:**
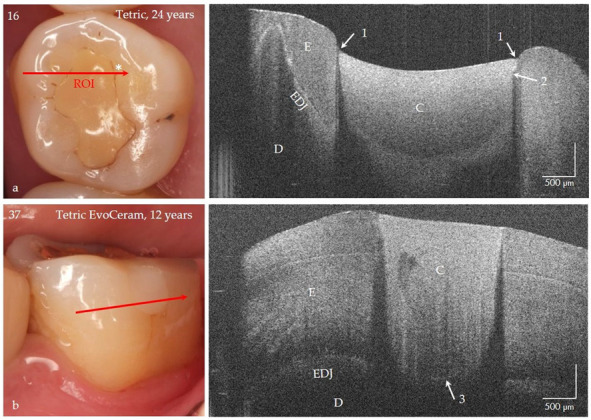
Class I and V composite restorations (C) in teeth 16 and 37 after 24 and 12 years of clinical function, imaged with OCT from the occlusal (**a**) and vestibular view (**b**). (**a**) The surface of the composite restoration (Tetric, adhesive #) shows negative steps at the restoration margins (arrows 1). The marginal discoloration (*) can be explained by the short interfacial marginal gap and not by caries adjacent to the restoration margin (secondary caries) (arrow 2, bright line). Beyond that the bonding interface is still intact after 24 years (no bright signal lines) and the material is homogeneous. (**b**) At the floor of the 12-year-old restoration (Tetric EvoCeram, adhesive #) a short interfacial gap (3) is indicated, the progression of which can be monitored. Compared to tooth 16, the restoration margins are flat, and the material exhibits inhomogeneities. *Enamel (E), dentin (D), the enamel-dentin junction (EDJ). The red arrows mark the section planes of the OCT cross-sectional images. The vertical scales are related to the refractive index n = 1.0 (see remark in Figure 4).*

**Figure 16:**
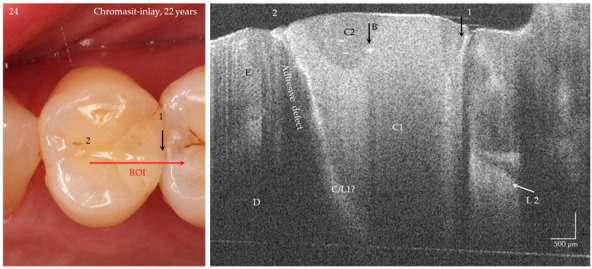
Chromasit^®^ inlay on tooth 24 after 22 years of clinical service. The OCT-B scan of the tooth-restoration interface shows an extended marginal and deep interfacial gap (position 2, bright signal line) without signs of secondary caries. At and beneath the enamel-dentin junction, a bright zone indicates a carious lesion at the tooth-composite contact zone or a cracked zone in the bonding composite (C/L 1?). Gap 1 is the gap between the teeth (short bright signal line). The restoration materials C1 (dentin color) and C2 (enamel color) contain single bubbles (B). A proximal lesion L 2 on tooth 25 mesial is visible, which could also be confirmed tactile (ICDAS, code 3) after OCT imaging (verification). *Composite (C), enamel (E), dentin (D). Refractive index n = 1.0 (see Figure 4).*

**Figure 17:**
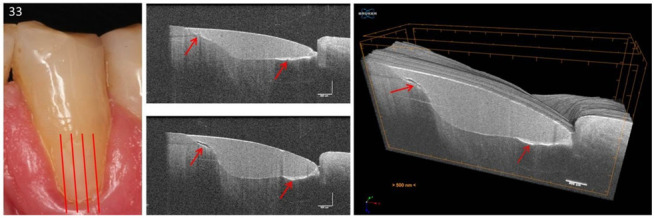
Tooth 33. 3D scan of a vestibular composite restoration. In the sequence of B-scans of the 3D image stack, restoration irregularities can be displayed, and their progress documented. An extended bond failure on dentin and numerous bubbles in the composite can be seen representing communicating tubes (red arrows). Such irregularities can, for example, lead to marginal or restoration discoloration and restoration loss. In clinical studies, these parameters are used to evaluate restoration systems [24]. *The vertical scales are related to refractive index n = 1.0.*

Based on the proven periods of up to 24 years that the composite restorations were in clinical function, the success or weaknesses of the adhesive technique and composite application could be illustrated (Figure 14a–f, Figure 15, Figure 16 and Figure 17). It was noticeable that the cavities are usually very irregularly coated with adhesive, whereby adhesive layers from a thickness of approx. 7 µm can be displayed (Figure 14d,e). In composite restorations, inhomogeneities of the material (Figure 15, Figure 16 and Figure 17), bubbles, incremental lines, various finely structured irregularities, and material defects were imaged (Figure 14, Figure 15, Figure 16 and Figure 17). There were also restorations with composite excesses (Figure 14a,c), and after years of clinical function, positive and negative steps were seen at the restoration margins (Figure 14a,e and Figure 15a) or the recession of the marginal gingiva (Figure 14d,f and Figure 17). By imaging interfacial adhesive defects (interfacial gaps) up to a depth of 2.5 mm, it was possible to display the failure of the tooth-composite bond. This is particularly true for the restoration margin (Figure 14b,f, Figure 15, Figure 16 and Figure 17). All shown structural features can be quantified, and their change can be monitored by repeated imaging.

## 4. Discussion

In this study, SD-OCT was used for the intraoral detection of proximal cavitated and non-cavitated carious lesions. For this purpose, an intraorally positionable OCT probe was developed to enable the already known potential of optical coherence tomography to be used on tooth surfaces that cannot be reached from extraorally. To the authors’ knowledge, this report is the first to show the clinical use of OCT for the detection of proximal caries lesions in comparison with the classical diagnostic procedures of visual inspection, bitewing radiography and fiber optic transillumination. Furthermore, it was investigated to what extent the intraoral OCT-probe can provide additional clinically relevant information for the evaluation of tooth-colored restorations under clinical conditions and especially on difficult to access tooth surfaces. All tooth surfaces, including those of the third molars, were included.

### 4.1. Diagnosis of Proximal Carious Lesions

Caries diagnosis of the proximal surfaces is difficult because the areas are often not visually accessible due to the close tooth contact and the gingival papilla and tooth separation cannot be established as a standard. In these situations, the combination of visual inspection and radiography are the classical standard methods for caries detection. Beyond that, FOTI is used in clinical practice as an easy-to-use and safe method to complement visual inspection. The non-invasive method is based on the transillumination of the hard tooth tissues by the light of the caries probe. Occlusally, carious regions appear as dark zones due to the different scattering and absorption of light in healthy and carious enamel and dentin. FOTI is recognized as a reliable method for the detection of anterior proximal caries and dentin caries in the posterior region with a higher sensitivity for dentin lesions compared to enamel lesions [10]. However, like classical intraoral radiography, this technique is not able to display surface cavitations [44].

The OCT probe enables the imaging of tooth surfaces from vestibular, occlusal, and oral directions of viewing and the display of the internal anatomical structures of the hard tooth tissues (first hypothesis). Variation of the angle of light incidence contributes to increasing the reliability of detection of (hidden) lesions that are difficult or impossible to detect by default, especially if they are located below the contact point [41]. Apart from the vestibular tooth surfaces of the third molars, all other surfaces can be imaged with the probe. The "blind spot" in the proximal zones of the molars results from the limited penetration depth at the enamel of 2.5 mm (central wavelength of 1310 nm), the diameter of the probe used, and the parameters of the imaging optics. The challenge is to avoid or minimize this non-imaging zone, e.g., by designing a probe with a more filigree tip and/or increasing the penetration depth by using a longer central wavelength. The parameters of the imaging optics must always be adapted to the specific requirements (resolution, field of view, contrast etc. [23]).

Since there is no gold standard for in vivo caries diagnosis, a comparative evaluation of the four methods was based on contingency tables. In particular, this approach reveals the differences between the methods used for evaluating a "healthy tooth surface". As in vitro, the histological investigation of tooth sections is considered the gold standard for the assessment of healthy and carious hard tooth tissues, non-invasive recording of cross-sectional images with OCT can be considered an appropriate analog for the detection of lesions up to a depth of 2.5 mm in in-vivo evaluation. This is especially true, as previous studies have validated OCT [41,42,45] and shown its high potential for early detection of enamel carious lesions [22,35,36,41,42,46]. In the OCT image stack, the maximum extension of a lesion can be determined, but also micro-cavitations can be resolved, which is not so simple to realize when investigating tooth sections in vitro using light microscopy.

While the dichotomous classification used here showed a high agreement of 94% between OCT and established methods (Figure 8a) for carious lesions, there was a clear difference in the surfaces assessed as sound (13%, low agreement). OCT generated a signal for demineralized enamel for 14 of the 16 surfaces that were classified as healthy with the methods introduced. In contrast, no signals were detected with OCT in three surfaces that were reproducibly visually assessed with score 1. In principle, OCT differentiated more strongly and reliably, whereas the classical methods did not detect very small defects at all. The second and third hypotheses can therefore be accepted. The very low agreement shows the extent of the expected inaccuracies in the assessment of initial enamel demineralisation and is consistent with the results of other studies [33]. It strongly supports the demands for more reliable techniques, especially for the detection of early caries, in order to give more space to caries prevention in the future [2,4].

OCT can also be a valuable decision support for advanced lesions if the extent of the lesion is to be assessed more precisely and subjected to monitoring or if cavitations are to be identified as a central decision cut-off for invasive interventions [41,47]. The high sensitivity of the OCT device system used and the high spatial resolution achieved with SD-OCT permits, in addition to the early detection of lesions, clearer discrimination of lesion bodies, and the imaging of micro-cavitations. The latter poses a particular challenge since cavitations can probably often only be made visible by varying the angle of light incidence in the OCT image. More research is needed to further clarify this point. This could also help to reduce the uncertainties in the use of the term micro-cavitation, as these can be displayed [44]. Furthermore, OCT can be used in situations where the extent of the carious lesions cannot be adequately confirmed by visual inspection or is underestimated during radiological examination [33]. Unclear visual findings or ambiguous radiolucencies extending into the dentin, which may indicate cavitation, could be verified by the higher spatial resolution (third hypothesis) [47].

### 4.2. Tooth-Colored Restorations

It is known that the durable bond between the restoration and the tooth, and in particular its peripheral sealing to prevent secondary caries, is of central importance [44,48]. Since OCT reveals interfacial adhesive defects, irregularities in the sealing of the cavity surface with adhesivesand in restorative materials, it can be used as a tool for optimizing the technique-sensitive steps of the adhesive technique (fourth hypothesis). One aspect that was demonstrated in this study should be the aim of further investigations: the irregular or slight coating of the cavities with adhesive. There is still great potential here if OCT diagnostics can be used to support and control the sealing of cavities with adhesive layers of appropriate thickness. The same applies to the failure-free application of the composites. By monitoring the polishing of the restoration composite, any surplus material can be avoided. Thus, discolored restoration margins can be minimized, which takes into account the recommended “guiding principles” of restoration management [44]. Of high clinical relevance, however, is the possibility of differentiating between discoloration of the restoration margins and secondary caries, which can prevent false-positive diagnoses and thus overtherapy (Figure 15a and Figure 16). The clinical evaluation of composite restorative systems can be supplemented by monitoring the restoration using OCT without the risk of radiation damage with a considerable gain in information [20,23,24,25,46,49]. This concerns, in particular, the assessment of the integrity of the tooth-composite interface. In clinical studies, differences in the performance of several systems became apparent immediately after placement of the fillings, which only became clinically apparent months later in the follow-up [24,25].

### 4.3. Methodological Criticism/Limitations of the Intraoral OCT Probe

This study compared four methods for the diagnosis of caries in 64 approximal tooth surfaces of a small study population. Significant differences between the methods were found when 22 to 13 surfaces per subject were assessed (Figure 7, probands 1, 3, 4). In contrast, no significant differences between the methods could be detected with the eight surfaces of proband 2. Empirically, this leads to the conclusion that, under the conditions of the study, 13 surfaces are sufficient to show differences between the methods. Since no reference standard was available, no statistical measures could be determined to show the diagnostic accuracy of the methods (specificity, sensitivity). In three clinical cases where treatment was necessary, the (blind) diagnosis OCT-score 1 could be confirmed in combination with performed dental treatments (Figure 11 and Figure 13). In the future, intraoral OCT must be validated using such clinical cases and on a broader study population. Furthermore, the simplifying dichotomization in presence and absence of a carious lesion/cavitation should be modified and the spectrum of different appearances of carious defects needs to be considered. For ethical reasons, however, in vivo invasive validation procedures are not applicable to teeth that are assumed to be healthy. Therefore, this has to be performed in vitro on artificial sets of teeth in a patient-equivalent simulation, which simultaneously allows the use of reference methods or by using construct validity in longitudinal in vivo settings. However, the validation of early carious enamel lesions, especially in difficult to access areas, will remain a clinical challenge as a diagnostic reference is currently lacking. Nevertheless, early lesions should be the focus of further development of diagnostic procedures, as only they allow early non-invasive intervention with longitudinal monitoring.

The further development of the evaluation of tooth-colored restorations with the intraoral OCT probe faces the similar challenges. Both in vivo and in vitro, the number of restorations as well as materials examined must be further increased. However, comprehensive clinical studies are already being carried out in this area, in which the benefits of OCT in the evaluation of composite restorations are being examined in more detail [20,23,24,25].

Finally, another aspect of OCT should not be ignored in future considerations. Imaging with self-explanatory real-time OCT image stacks offers the chance to communicate intervention considerations to the patient in a simple way and to involve him or her in the treatment process.

## 5. Conclusions

The hand-held OCT probe may be a valuable non-invasive supplement for intraoral caries diagnosis and the evaluation of non-metallic adhesively luted restorations. It can complement the diagnostic methods already introduced, especially in the currently difficult detection and assessment of early and very early enamel caries lesions, as well as in the detection of approximal cavitations. Furthermore, the probe offers possibilities in quality management, e.g., in the preparation of tooth-coloured restorations, and can generally contribute to a fact-based participative communication between dentists and patients.

## Figures and Tables

**Table 1:** Parameters of the optical coherence tomography (OCT) probe (IOS: OCTH-1300NR-SP6).

Central wavelength ± bandwidth	1310 nm ± 107 nm
Power on sample	2.3 mW
Sensitivity	≤106 dB
Imaging depth	2.5 mm
Pixels per A-scan	727
NA	0.011
Axial resolution (water)	5.5 µm
Lateral resolution (spot size)	11.5 µm
A-scan	28 kHz
B-scan	10 Hz
FOV (maximal)	≤8 mm × 8 mm
Angulation	90°

## Supplementary Materials

The following are available online at https://www.mdpi.com/2077-0383/9/10/3257/s1, Figure S1: Workflow.
