# Supplementary figures and images for: An Intraoral OCT Probe to Enhanced Detection of Approximal Carious Lesions and Assessment of Restorations
Source: J Clin Med. 2020 Oct 12;9(10):3257. doi: 10.3390/jcm9103257 (PMC7600310; doi:10.3390/jcm9103257)

**Figure S1. Work Flow**

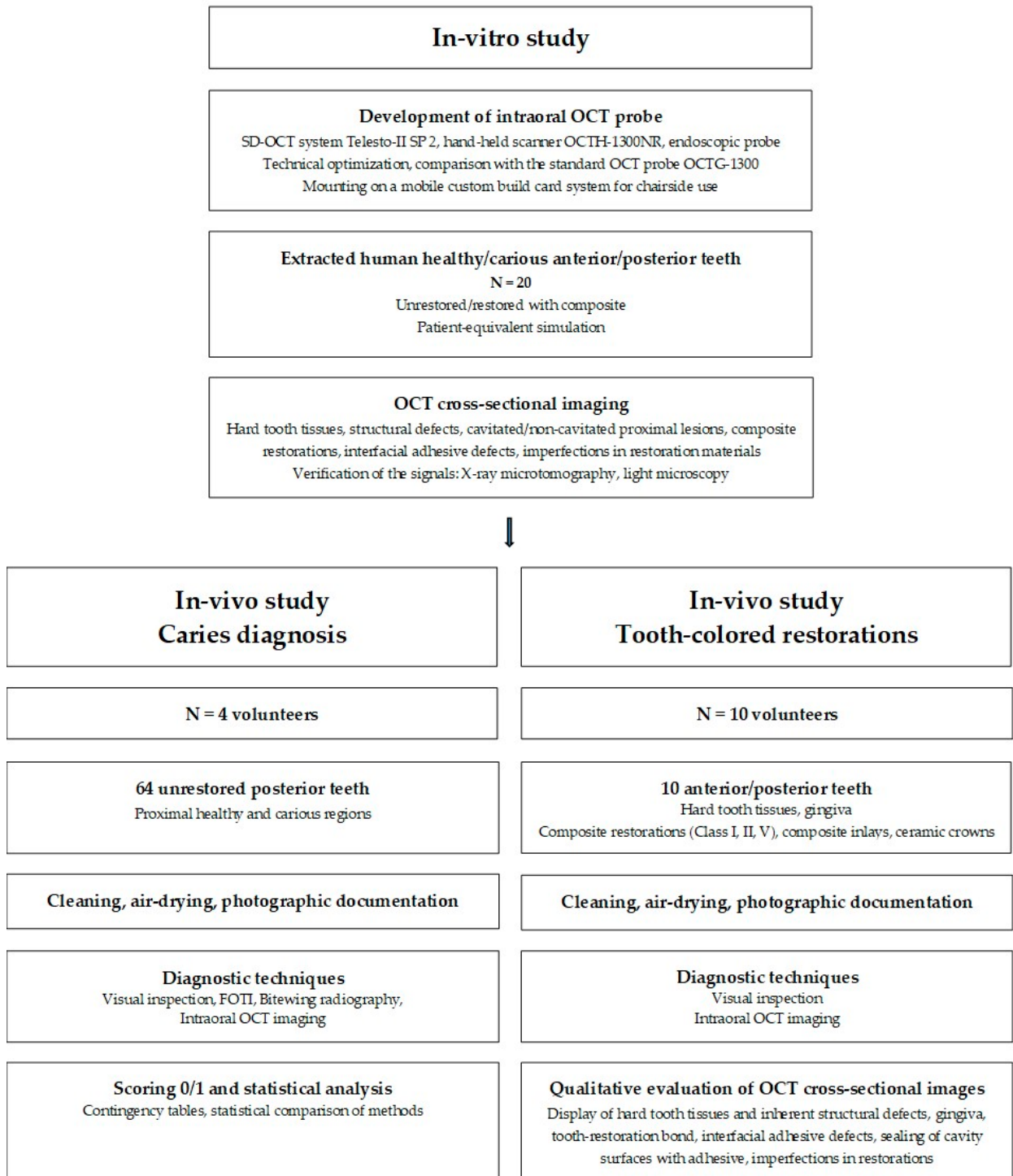

Supplement: Supplementary file 1 [file jcm-09-03257-s001.pdf]
